# 

**Published:** 1895-08

**Authors:** 


### CONTRIBUTORS TO VOLUME XXXV.

---

Abbott, A. C. . . . . . . . . . . . . . . . . . . . . . . . . Philadelphia.
Baker, C. O . . . . . . . . . . . . . . . . . . . . . . . . . Auburn.
Bartlett, F. W. . . . . . . . . . . . . . . . . . . . . . . . Buffalo.
Bender, Ida C. . . . . . . . . . . . . . . . . . . . . . . . . Buffalo.
Bennett, Arthur G. . . . . . . . . . . . . . . . . . . . . . . Buffalo.
Benninghoff, G. E. . . . . . . . . . . . . . . . . . . . . . . Bradford.
Bissell, William G. . . . . . . . . . . . . . . . . . . . . . . Buffalo.
Carroll, Jane W. . . . . . . . . . . . . . . . . . . . . . . . Buffalo.
Carroll, Evangeline . . . . . . . . . . . . . . . . . . . . . . Buffalo.
Crego, Floyd S. . . . . . . . . . . . . . . . . . . . . . . . . Buffalo.
Conklin, William L. . . . . . . . . . . . . . . . . . . . . . . Rochester.
Cronyn, John . . . . . . . . . . . . . . . . . . . . . . . . . Buffalo.
Crothers, T D. . . . . . . . . . . . . . . . . . . . . . . . . Hartford.
Daggett, B H. . . . . . . . . . . . . . . . . . . . . . . . . . Buffalo.
Daniels, C. M. . . . . . . . . . . . . . . . . . . . . . . . . Buffalo.
D'Estes, Debout. . . . . . . . . . . . . . . . . . . . . . . . Paris.
Diehl, Alfred E. . . . . . . . . . . . . . . . . . . . . . . . Buffalo.
Dickinson, Mary E. . . . . . . . . . . . . . . . . . . . . . . Dansville.
Dorr, Samuel G. . . . . . . . . . . . . . . . . . . . . . . . . Buffalo.
Doyle, Gregory . . . . . . . . . . . . . . . . . . . . . . . . Syracuse.
Dunham, Sydney A. . . . . . . . . . . . . . . . . . . . . . . . Buffalo.
Elsner S. L . . . . . . . . . . . . . . . . . . . . . . . . . . Rochester.
Frear, Jane N. . . . . . . . . . . . . . . . . . . . . . . . . Buffalo.
Frederick, C. C. . . . . . . . . . . . . . . . . . . . . . . . Buffalo.
Frye, Maud J . . . . . . . . . . . . . . . . . . . . . . . . . Buffalo.
Gram, Franklin C. . . . . . . . . . . . . . . . . . . . . . . . Buffalo.
Hayd, Herman E. . . . . . . . . . . . . . . . . . . . . . . . . Buffalo.
Hall, A. L. . . . . . . . . . . . . . . . . . . . . . . . . . . Fair Haven.
Hauenstein, John . . . . . . . . . . . . . . . . . . . . . . . Buffalo.
Hill, Julian P. . . . . . . . . . . . . . . . . . . . . . . . . Buffalo.
Himmelsbach, G. A. . . . . . . . . . . . . . . . . . . . . . . Buffalo.
Hopkins, Henry R. . . . . . . . . . . . . . . . . . . . . . . . Buffalo.
Howell, S. Y. . . . . . . . . . . . . . . . . . . . . . . . . . Buffalo.
Hubbell, A. A. . . . . . . . . . . . . . . . . . . . . . . . . Buffalo.
Huntley, Mary M . . . . . . . . . . . . . . . . . . . . . . . . Buffalo.
Jones, Wm. B. . . . . . . . . . . . . . . . . . . . . . . . . . Rochester.
Krauss, Wm. C. . . . . . . . . . . . . . . . . . . . . . . . . Buffalo.
Kuhlman, Helene . . . . . . . . . . . . . . . . . . . . . . . . Buffalo.
Lytle, Albert T. . . . . . . . . . . . . . . . . . . . . . . . Buffalo.
Lothrop, Thomas . . . . . . . . . . . . . . . . . . . . . . . . Buffalo.
Mann, Matthew D. . . . . . . . . . . . . . . . . . . . . . . . Buffalo.
Mathews, Joseph M. . . . . . . . . . . . . . . . . . . . . . . Louisville.
Matzinger, H. G. . . . . . . . . . . . . . . . . . . . . . . . Buffalo.

Maloney, F. M . . . . . . . . . . . . . . . . . . . . . . . . . . Rochester.
Macbeth, A H. . . . . . . . . . . . . . . . . . . . . . . . . . . Buffalo.
Miller, John A. . . . . . . . . . . . . . . . . . . . . . . . . . . Buffalo
Moehlau, F. G. . . . . . . . . . . . . . . . . . . . . . . . . . . Buffalo.
Morris, Robert T. . . . . . . . . . . . . . . . . . . . . . . . . New York.
Mulford, Henry J. . . . . . . . . . . . . . . . . . . . . . . . . Buffalo.
Mynter, Herman . . . . . . . . . . . . . . . . . . . . . . . . . Buffalo.
Park, Roswell . . . . . . . . . . . . . . . . . . . . . . . . . . Buffalo.
Parmenter, John . . . . . . . . . . . . . . . . . . . . . . . . . Buffalo.
Palmer, George M. . . . . . . . . . . . . . . . . . . . . . . . . Warsaw.
Pitkin, John T. . . . . . . . . . . . . . . . . . . . . . . . . . Buffalo.
Pohlman, Julius . . . . . . . . . . . . . . . . . . . . . . . . . Buffalo.
Potter, William Warren . . . . . . . . . . . . . . . . . . . . . Buffalo.
Possanner, Gabrielle Baronin . . . . . . . . . . . . . . . . . . Vienna.
Putnam, James Wright . . . . . . . . . . . . . . . . . . . . . . Buffalo.
Peterson, Frederick . . . . . . . . . . . . . . . . . . . . . . New York.
Randall, Lillian Craig . . . . . . . . . . . . . . . . . . . . . Buffalo.
Renner, W. Scott . . . . . . . . . . . . . . . . . . . . . . . . Buffalo.
Satterlee, R. H. . . . . . . . . . . . . . . . . . . . . . . . . Buffalo.
Stanton, William . . . . . . . . . . . . . . . . . . . . . . . Varysburg.
Stephenson, F. H. . . . . . . . . . . . . . . . . . . . . . . . Syracuse.
Sellstedt, Lars Gustav, Esq. . . . . . . . . . . . . . . . . . . Buffalo.
Smith, Eugene A. . . . . . . . . . . . . . . . . . . . . . . . . Buffalo.
Snow, Irving M. . . . . . . . . . . . . . . . . . . . . . . . . . Buffalo.
Snow, Sargent F. . . . . . . . . . . . . . . . . . . . . . . . . Syracuse.
Stockton, Charles G. . . . . . . . . . . . . . . . . . . . . . . Buffalo.
Storck, Edward . . . . . . . . . . . . . . . . . . . . . . . . . Buffalo.
Tait, Lawson, Esq. . . . . . . . . . . . . . . . . . . . . . . Birmingham.
Toms, S. W. S. . . . . . . . . . . . . . . . . . . . . . . . . . Bellport.
Vandenbergh, Bina Potter- . . . . . . . . . . . . . . . . . . . Dansville,
Wende, Ernest . . . . . . . . . . . . . . . . . . . . . . . . . . Buffalo.
Wende, G. W. . . . . . . . . . . . . . . . . . . . . . . . . . . . Buffalo.
Whipple, Electa B. . . . . . . . . . . . . . . . . . . . . . . . . Buffalo.
Wyckoff, C. C. . . . . . . . . . . . . . . . . . . . . . . . . . . Buffalo.
Buffalo Academy of Medicine.
Medical Association of Central New York.
Medical Society of the County of Chautauqua.
Medical Society of the County of Erie.
National Confederation of State Medical Examining Boards.

